# Synopsis of valid species-group taxa for freshwater Gastropoda recorded from the European Neogene

**DOI:** 10.3897/zookeys.435.8193

**Published:** 2014-08-15

**Authors:** Thomas A. Neubauer, Andreas Kroh, Mathias Harzhauser, Elisavet Georgopoulou, Oleg Mandic

**Affiliations:** 1 Geological-Paleontological Department, Natural History Museum Vienna, 1010 Vienna, Austria

**Keywords:** Freshwater mollusks, species list, taxonomy, online sources, Miocene, Pliocene

## Abstract

Here we present a complete list of all valid species-group taxa of freshwater gastropods reported from Miocene and Pliocene deposits in Europe. The last comparable work dates back to the 1920s and covered about 1,600 names. The extensive literature research underlying the present work revealed considerable changes in the taxonomic and systematic frameworks of Neogene freshwater gastropods and yielded a total number of 2,156 accepted taxa. Each taxon is accompanied by a full citation of its first description; where the information is available, page number and illustration reference are provided. First descriptions available as open-access full-text sources on the web were linked via hyperlink to the first page of the publication.

## Introduction

The latest available synopsis for continental gastropods of the Paleogene and Neogene of Europe is the over 3000-page-thick Fossilium Catalogus of Wenz (1923–1930), who managed to include data of almost all the literature published before 1923. For the freshwater Neogene (i.e., Miocene and Pliocene, 23.03–2.588 Ma, Gradstein et al. 2012) this resulted in a number of about 1,600 accepted species-group names. Since then numerous new papers and monographs have been published, which introduced new names, new combinations, and/or synonymized others and re-arranged many of the systematic classifications (e.g., Pavlović 1927, 1931; Jekelius 1932, 1944; Wenz 1938–1944; Papp 1953, 1954; Sauerzopf 1953; Bartha 1954, 1956; Schlickum 1964, 1976; Gillet and Marinescu 1971; Roshka 1973; Schütt 1976; Willmann 1981; Lubenescu and Zazuleac 1985; Anistratenko 1993; Rust 1997; Harzhauser and Binder 2004; Harzhauser et al. 2002; Gozhik and Datsenko 2007; Neubauer et al. 2011, 2013a, 2013b, 2014a, 2014b). Consequently, the inventory has changed substantially since Wenz.

This data paper presents a list of all accepted species-group names of the European Neogene freshwater gastropods, each with the current generic combination and reference to the first description. In many cases we referred to the latest available combination, but did not aim for a critical evaluation of these; this contribution is not meant as taxonomic revision.

## Methods

The presented data derive from 459 literature sources. Nearly all of the first descriptions have been seen and verified by the authors (97.4%). We checked for correct spelling and nomenclatural validity (to exclude nomina nuda). Where feasible, we tried to include the full citation with indication of page number and illustrations (if present). Despite much effort it was not possible to acquire all of the mentioned publications. In such cases reference and indication of pages/illustrations were taken from Wenz (1923–1930) or other reliable sources where possible. For 9 taxa (0.4%) precise data is missing; for 11 taxa (0.5%) information is incomplete because the respective publication was only partially accessible to the authors (Gozhik and Prysjazhnjuk 1978). An absent indication of illustration does not necessarily imply that there is no depiction in the original publication. Additionally hyperlinks to open-access online sources (e.g., Biodiversity Heritage Library, Internet Archive, Digital Library of the State Museum of Upper Austria, Gallica – Bibliothèque nationale de France, Google Books) are included for many of the publications.

## Data setup

The list contains 2,156 accepted species-group taxa recorded for the Miocene and Pliocene. Since stratigraphical boundaries changed in the past decades and the age attributions of many gastropod-bearing localities have been revised since then, many taxa originally recorded for "Miocene" or "Pliocene" localities have been shown to belong to earlier or younger strata and vice versa. Although we tried to find out the most recent and accurate age attributions for the localities containing the relevant taxa, there are still doubtful cases where species may prove not to derive from Neogene sediments. Erroneous records of extant species in Neogene deposits were not considered herein.

It was not possible to trace the correct authorship for the species *Melanopsis atanasiui*, *Caspia dacica*, and *Prososthenia pertica*. The publications mentioned in the species list (Pană 1989; Pană et al. 1981) probably display the first descriptions, but erroneously cite wrong authorships (maybe referring to unpublished manuscripts). None of both works contain any reference to a publication matching the given authority, nor do they list compatible records in synonymy lists. For these three cases the probably correct authorships are stated; those given by the respective authors are included in square brackets.

The dataset includes the following fields:

**Taxon:** Fully spelt species-group name in the current combination, variably with indication of subgenus if stated that way in the literature

**Genus:** Genus name; again, sometimes with indication of subgenus

**Species/subspecies:** Epithet of species and subspecies, if available; given in its currently accepted combination and rank

**Authority:** Authorship and year of the taxon; in parentheses when not in original combination

**Source:** Full reference to the first description of the taxon

**Page/Illustration:** Indication of page number and illustration, if available

**Seen:** To indicate if the authors have personally checked the publication (v – seen, p – information partially available, n – not seen)

**Source hyperlink:** Hyperlink to first page of reference, if available

Although much time was spent to acquire all the relevant literature, this list may be incomplete. Moreover, not every described species is incorporated here, since the list only includes accepted names. Unaccepted names, such as primary homonyms, junior synonyms, nomina nuda, nomina dubia, or nomina inquirenda are not covered. For those older than 1923 see Wenz (1923–1930). Readers are invited to contact the authors for cases, where names – especially those thought to refer to accepted species-group taxa – are neither found in the here presented species list nor in Wenz or other sources.

Where a species contained one or several subspecific taxa (or they are currently ranked as such), the nominal subspecies was excluded from the list, so as not to overstate the actual number of accepted names.

## Supplementary material

Supplementary material 1 List of valid species-group taxa for Neogene freshwater Gastropoda of Europe doi: 10.3897/zookeys.435.8193.app1 Data type: Table (Microsoft Excel 97-2003 format) Explanation note: For details see chapter on data setup above. This dataset is made available under the Open Database License (http://opendatacommons.org/licenses/odbl/1.0/). The Open Database License (ODbL) is a license agreement intended to allow users to freely share, modify, and use this Dataset while maintaining this same freedom for others, provided that the original source and author(s) are credited.Thomas A. Neubauer, Andreas Kroh, Mathias Harzhauser, Elisavet Georgopoulou, Oleg Mandic
